# Salvianolic Acid A Inhibits OX-LDL Effects on Exacerbating Choroidal Neovascularization via Downregulating CYLD

**DOI:** 10.1155/2017/6210694

**Published:** 2017-09-01

**Authors:** Ke Mao, Wanting Shu, Libin Liu, Qing Gu, Qinghua Qiu, XingWei Wu

**Affiliations:** Department of Ophthalmology, Affiliated First People's Hospital, Shanghai Jiao Tong University, Shanghai, China

## Abstract

**Backgrounds:**

Age-related macular degeneration is closely related to lipid oxidation, while relationship between OX-LDL and choroidal neovascularization is unclear. Recently, cylindromatosis is proved to regulate angiogenesis. However, its role in CNV progression remained unclear. Salvianolic acid A is widely used in vascular diseases. We investigated the relationship between OX-LDL and CNV and explore antineovascularization mechanism of Sal A.

**Methods:**

C57BL6/J mice were randomized into four groups and injected with PBS or OX-LDL, together with Sal A for one week. CNV was induced by laser; CNV severity was analyzed by fundus fluorescein angiography, H&E staining, and choroid flat mount after 1 week. In in vitro experiments, ARPE-19 and HUVECs were cultured with OX-LDL (with or without Sal A) for 48 hours. Angiogenic proteins, cell junction integrity, and tube formation were measured. *CYLD* siRNA and specific inhibitors were used to explore mechanisms of CYLD in promoting OX-LDL-induced CNV progression.

**Results:**

OX-LDL promoted laser-induced CNV volume by increasing VEGF, PDGF, and CYLD levels. Sal A antagonized OX-LDL effects and restrained CNV progression by decreasing VEGF/PDGF/CYLD, increasing antiangiostatin levels, and promoting P62-CYLD-TRAF6 interaction.

**Conclusions:**

We demonstrated oxidation damage exacerbates CNV progression, and Sal A could be a clinical therapeutic reagent to exudative AMD.

## 1. Introduction

Choroidal neovascularization (CNV), the hallmark of exudative age-related macular degeneration (AMD), is responsible for approximately 90% of cases of severe vision loss caused by AMD [1].

CNV is a pathological angiogenesis arising from choriocapillaris, resulting in the accumulation of fluid within the retina and subretinal space [2]. Pharmacotherapy by intravitreous administration of VEGF inhibitors has been used regularly while the curative effect is unstable [3]. The pathology of exudative AMD is complicated and associated with multiple pathologic factors including photooxidative stress, complement activation, cellular senescence, and microbial assault [4]. Among all the proposed factors, oxidative stress has multieffects and plays a critical role in cardiovascular diseases and AMD [5]. Carbohydrates, membrane lipids, proteins, and nucleic acids are all vulnerable to oxidative damage and contribute to AMD progression [6].

Changes in lipid profile in terms of total cholesterol (TC), triglycerides (TG), low-density lipoprotein (LDL), and high-density lipoprotein (HDL) have been reported in AMD progression [7]. LDL is susceptible to oxidation, resulting in the formation of oxidized low-density lipoprotein (OX-LDL) [8]. Oxidized lipoproteins have been detected in CNV membranes from AMD patients [9]. Our previous studies established an animal model to study the biological effects of high circulating serum LDL on retinal pigment epithelium (RPE) and demonstrated that OX-LDL leads to RPE cell apoptosis and inflammation, which indicated the mechanism of nonexudative AMD [10, 11]. However, it remained unclear whether OX-LDL affects CNV progression and is worth studying.

Salvianolic acid A (Sal A) is the active monomer extracted from *Salvia miltiorrhiza* Bunge (Danshen), which is a traditional Chinese medicine and has been administrated in AMD clinically [12]. Sal A is a phenolic carboxylic acid derivative which presents a variety of pharmacological functions including anti-inflammation, antioxidation, and antiplatelet effects [13]. We found that Sal A protects RPE cells from OX-LDL-induced inflammation, while it remains largely unknown whether Sal A could repress CNV progression.

Cylindromatosis (CYLD) is a tumor suppressor that regulates signaling pathways by acting as a deubiquitinating enzyme [14]. CYLD regulates diverse biological processes including cell proliferation, survival, migration, immune responses, osteoclastogenesis, and spermatogenesis [15]. Recently, CYLD was identified as a potential modulator of vascular formation [16]. However, whether CYLD involves in the pathological process of CNV has not been studied before.

Therefore, this study was undertaken to assess the biological importance of OX-LDL and its relationship with CYLD in modulating CNV progression, meanwhile proved the potential therapeutic value of Sal A.

## 2. Materials and Methods

### 2.1. Animals and Reagents

Male wild-type C57BL/6J mice (75–100 g) were purchased from the Shanghai Laboratory Animal Center of the Chinese Academy of Sciences and used for a pathologic study. Animals were kept under a 12-hour dark/light circle. This study was approved by the Institutional Animal Care and Use Committee (IACUC) at the medical academy of Shanghai Jiao Tong University. All animal experiments were performed in accordance with the guidelines of the ARVO Statement for the Use of Animals in Ophthalmic and Vision Research. Human OX-LDL was purchased from AppliChem (Darmstadt, Germany), and OX-LDL quality was detected by electrophoretic methods. Sal A was purchased from Nanjing Guangrun Biochemical Company (Nanjing, China). Forty mice were randomized into 4 groups: PBS, OX-LDL (3 mg/kg body weight), Sal A (10 mg/kg body weight), and OX-LDL (3 mg/kg) + Sal A (10 mg/kg) (*n* = 10 per group). Each group was injected with PBS or OX-LDL in the vein for 7 days; Sal A was intraperitoneally injected 3 hours before OX-LDL or PBS administration once per day.

### 2.2. Serum Lipid Analyses

The serum of mice in each group was collected, and we measured serum lipoprotein levels after consecutive injections of OX-LDL for 7 days. Concentrations of serum total cholesterol and OX-LDL cholesterol were measured with the commercial ELISA kits (Kmaels, Shanghai, China) and an automated biochemistry platereader (Olympus AU600, Tokyo, Japan).

### 2.3. Induction of Choroidal Neovascularization (CNV)

CNV was induced by laser photocoagulation as described previously [17–19]. To assess CNV volumes, 3-4 spots of laser photocoagulations (parameters: 532 nm laser; power, 130 mW; duration, 100 ms; diameter, 50 *μ*m; Novus Verdi, Coherent Inc., Santa Clara, CA, USA) were placed in the fundus of each eye. The laser spots were created around the optic nerve using a slit-lamp delivery system, a coverslip was used to allow viewing of the posterior pole of the eye. The morphologic endpoint of the laser injury was the appearance of a cavitation bubble, which is a sign of Bruch's membrane disruption.

### 2.4. Fundus Fluorescein Angiography (FFA) Imaging and Grading

Fundus fluorescein angiography was performed 7 days postlaser by intraperitoneal injection of 50 *μ*L of 25% fluorescein sodium (Alcon, Fort Worth, TX, USA). Fundus images were taken using a digital fundus camera (Model TRC 50 IA; Topcon, Paramus, Japan). FFA was qualitatively and quantitatively evaluated by two blind groups of observers. The laser-induced lesions were graded based on the observed fluorescein leakage and then divided into the following four categories as described by Semkova [20]. Volumes of grade 4 laser spots were evaluated by image J software.

### 2.5. CNV Volume Analysis

Choroidal flat mount preparation, staining, and imaging were undertaken as previously described [21]. Briefly, eyes were enucleated at 7 day after laser injury and immediately fixed for 1 h in a solution of 4% paraformaldehyde in phosphate-buffered saline (PBS; 9 g/L NaCl, 0.232 g/L KH_2_PO4, and 0.703 g/L Na^2^HPO4; pH 7.3). The anterior segment and crystalline lens were removed, and the retinas were detached and separated from the optic nerve head with a pair of fine-curved scissors. The remaining eye cups were washed with cold ICC buffer (0.5% BSA, 0.2% Tween 20, and 0.05% sodium azide) in PBS. Next, a 1 : 500 dilution of isolectin B4 (Sigma-Aldrich, USA) and 1 : 1000 dilution of CD31 (Abcam, Cambridge, UK) were incubated at 4°C overnight and then washed with cold PBS buffer. A 1 : 1000 dilution of fluorescence-conjugated secondary antibody (Abcam, Cambridge, UK) was incubated for 1 hour and then washed with cold PBS buffer. Radial cuts were made toward the optic nerve head, and the sclera-choroidal/RPE complexes were flat mounted, covered, and sealed. Image J software was used to analyze fluorescence images. The summation of the whole stained area in each section multiplied by the distance between sections (1 *μ*m) was used as an index for the CNV lesion volume. The volumes of the all lesions in each eye were averaged and considered as an *n* = 1 for statistical analysis.

### 2.6. Hematoxylin and Eosin (HE) Staining and Immunofluorescence

Histopathological analysis was performed as described previously [22]. Mice were killed, eyes were enucleated 7 days after laser injury, and eyecups were fixed in paraformaldehyde at 4°C for 24 h. The fixed tissues were embedded in paraffin, serially sectioned at 3 *μ*m, and stained with hematoxylin and eosin (HE). Serial slices of each CNV were examined and digitized using a light microscope (Olympus Corporation, Tokyo, Japan). CNV thickness was measured vertically from the adjacent RPE layer to the top of the CNV, and CNV length was measured horizontally maximizing the distance of CNV using Image J software, which was expressed in *μ*m. For CYLD immunofluorescence, CNV sections were incubated overnight at 4°C in rabbit antihuman CYLD monoclonal antibody (Abcam, Cambridge, UK). Secondary antibodies were conjugated to AlexaFluor488 (Abcam, Cambridge, UK). The fluorescence intensity of CYLD was measured by Image J software.

### 2.7. Cell Culture

The human RPE cell line (ARPE-19) and human umbilical vein endothelial cells (HUVECs) were cultured as previously described [11]. ARPE-19 cells were grown to 70–80% confluence and placed in a serum-free medium (SFM) for 24 hours before treatments. Cells were then randomized into SFM, OX-LDL (100 mg/L), and OX-LDL + Sal A (5/50 *μ*M) for 48 hours. Cells were pretreated with Sal A 3 hours before OX-LDL stimulation. For ERK and PI3K/mTOR inhibitor treatment, ARPE-19 cells were pretreated with FR 180204 (10 *μ*M, Selleck, USA), LY294002 (1 mΜ, Darmstadt, Germany), or rapamycin (100 nM, Sigma-Aldrich, USA) for 1 hour, followed by Sal A (50 *μ*M) for 3 hours, then stimulated with OX-LDL (100 mg/L) for 72 hours.

### 2.8. CYLD RNAi

To silence *CYLD* gene in ARPE-19 and HUVEC cells, the specific mixture of three preselected siRNA duplexes to target different sequences of the human *CYLD* gene was utilized according to the manufacturer's instructions. The siRNA was mixed with Opti-MEM (Invitrogen, Carlsbad, CA) and Lipofectamine®3000(ThermoFisher Scientific, USA) to form the transfection complex, prior to addition to culture medium. Nonsilencing siRNA (SI03650325, Qiagen) with the same concentration was used as a negative control. The efficiency of gene silencing was determined by western blot 24 hours after the treatments.

### 2.9. Western Blot and Immunoprecipitation

Western blot was accomplished as described before [11]. Vascular endothelial growth factor (VEGF), platelet-derived growth factor (PDGF), pigment epithelium -derived factor (PEDF), P62, and antiangiostatin monoclonal antibodies were purchased from Abcam (Cambridge, UK). TRAF6 and CYLD monoclonal antibodies were purchased from CST (Boston, USA). *β*-actin was used as a loading control for each lane. Each indicated band was quantified and normalized to the corresponding loading control through Image J software. Immunoprecipitation was performed as follows: Antibodies against TRAF6 were used to precipitate proteins from cell lysis in the presence of 20 *μ*L protein A/G beads (Santa Cruz Biotechnology, Santa Cruz, CA, USA) overnight at 4°C. Protein complexes were washed 4 times with lysis buffer and then incubated at 95°C for 5 minutes. P62, CYLD, and ubiquitin proteins were resolved by western blot analysis.

## 3. Immunocytochemistry

As previously described [23], ARPE-19 cells were cultured on a 16-well glass slide. After treatment, cells were fixed for 20 minutes with 4% paraformaldehyde and permeabilized for one hour at room temperature with 10% goat serum and 0.1% Triton X-100 in PBS. ARPE-19 cells were then incubated with rabbit anti-human monoclonal antibodies directed against ZO-1 and CYLD (Abcam, Cambridge, UK) at 4°C overnight. The slides were then washed with PBS. Secondary antibodies were added to the slides for 1 hour. Slides were then rinsed with PBS, coated with mounting media containing DAPI, covered, and examined by fluorescent microscopy (Olympus, Japan).

### 3.1. ELISA

VEGF measurement after silencing *CYLD* gene was performed as described previously [24]. In brief, VEGF concentrations in ARPE-19 cell culture mediums were measured using a related human ELISA kit (R&D Systems, Minneapolis, USA) following the manufacturer's instructions. Serial dilutions of recombinant human VEGF were included in all assays to serve as standards. Triplicate evaluations were performed for each sample.

### 3.2. Real-Time PCR

Total RNA from the choroid of C57 mice were isolated using TRIzol reagent (Life Technologies, USA). cDNA synthesis was accomplished with First-Strand Synthesis System (TaKaRa Bio, Shiga, Japan). cDNA was amplified using the SYBR® Green PCR Master Mix Reagent (BIOTNT, Shanghai, China) on viia™ 7 Real-time PCR System (ABI, USA) as follows: 95°C, 5 min; followed by 35 amplification cycles (95°C for 5 s; 60°C for 30s). Primer sequences used were the following: *β*-actin, forward 5′-CCT CTA TGC CAA CAC AGT 3′ and reverse 5′-AGC CAC CAA TCC ACA CAG 3′; CYLD, forward 5′-AAT GTG TCC CTG CCC TAC CTA 3′ and reverse 5′-CTC GTC CCT ACT CTG CCA CTT 3′; PEDF, forward 5′-GAG GAC AGG ACC GTG AGA GT 3′ and reverse 5′-GGG CAG GAA GAA GAT GAT G 3′;VEGF, forward 5′-GCA AGA GAA GAC ACG GTG GT 3′ and reverse 5′-CAG GAG GTG GGG TAA GGA G 3′; PDGF, forward 5′-CAG TGT CCG TTT GTT CAG TG 3′ and reverse 5′-TGG TTT TGT TTT CGC TCT CT 3′; and angiostatin, forward 5′-CCT TGG TGC TAC ACT ACA GA 3′ and reverse 5′-GGA GAT TTT GCC CTC ATA C 3′. mRNA expression was normalized to the endogenous reference gene *β*-actin. Specific primers were produced by BIOTNT Company (Shanghai, China), and relative quantification was achieved by the comparative 2^−ΔΔct^ method as described [25].

### 3.3. Statistical Analysis

The experimental data are expressed as mean ± SEM in figures and mean ± SD in tables. Group means were compared by a one-way analysis of variance with the use of the GraphPad Prism 4.0 software system (GraphPad, San Diego, CA), and the statistical software program SigmaPlot v.10.0 software. *P* value less than 0.05 is considered to be significant.

## 4. Results

### 4.1. OX-LDL Increases Serum Lipoprotein Levels and Sal A Pretreatment Significantly Decreases Lipoprotein Levels

To investigate the effects of OX-LDL and Sal A injection on circulating lipoprotein level, we examined serum OX-LDL cholesterol and total cholesterol concentrations in C57 mice. Both OX-LDL and total cholesterol levels were significantly increased in the OX-LDL group compared with PBS group. And these OX-LDL-induced increases were significantly inhibited when pretreated with Sal A (see Table 1).

### 4.2. OX-LDL Increases Laser-CNV Volumes in C57 Mice, and Sal A Significantly Decreases CNV Volumes

**Figure 1:**
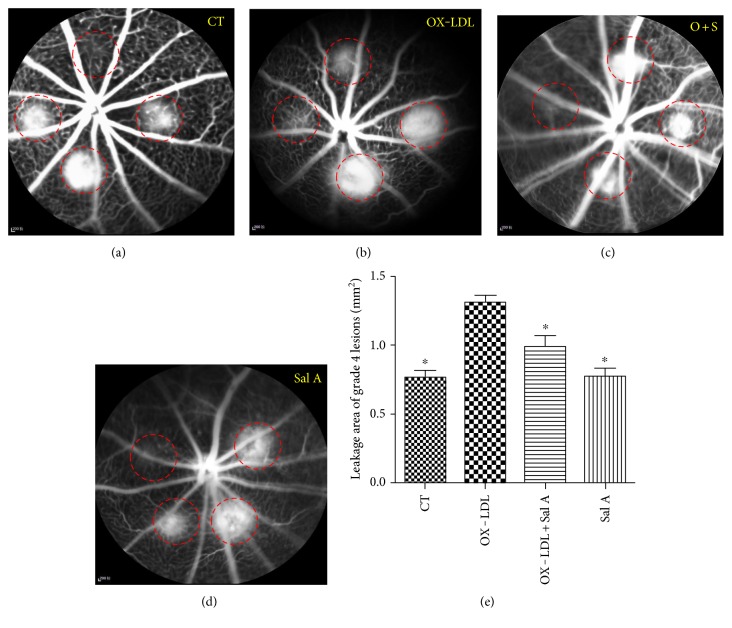
OX-LDL increases laser-induced CNV volume and leakage areas, and Sal A pretreatment antagonizes effects of OX-LDL. C57 mice were divided into PBS, OX-LDL, Sal A, and OX-LDL + Sal A groups. Each group was injected with PBS or OX-LDL or PBS administration. (a–d) Representative fluorescein angiograms 7 days postphotocoagulation showing late-phase leakages beyond borders. (a) Control group; (b) OX-LDL-injected group; (c) OX-LDL + Sal A-injected group; (d) Sal A-injected group. (e) Leakage areas of grade 4 lesions on the late phase of flourescein angiograms in each group. Leakage areas of grade 4 CNV lesions were measured by tracing the borders of fluorescein leakage using Image J software. Data were expressed as mean ± SEM. OX-LDL injection significantly increased a leakage area compared with the PBS group, and the OX-LDL + Sal A group had a smaller leakage area than the OX-LDL group (^∗^*P* < 0.05 versus the OX-LDL group).

Next, we studied the effect of OX-LDL and Sal A on CNV volume in vivo. FFA examination revealed CNV leakage in each group. Results in Figures 1()–1() showed that OX-LDL treatment significantly increased incidence and leakage areas of grade 4 lesion compared with the PBS group, while fluorescence leakage severity was significantly reduced in the Sal A + OX-LDL group when compared with the OX-LDL group. The grading of laser lesions in all groups is shown in Table 2.

**Figure 2:**
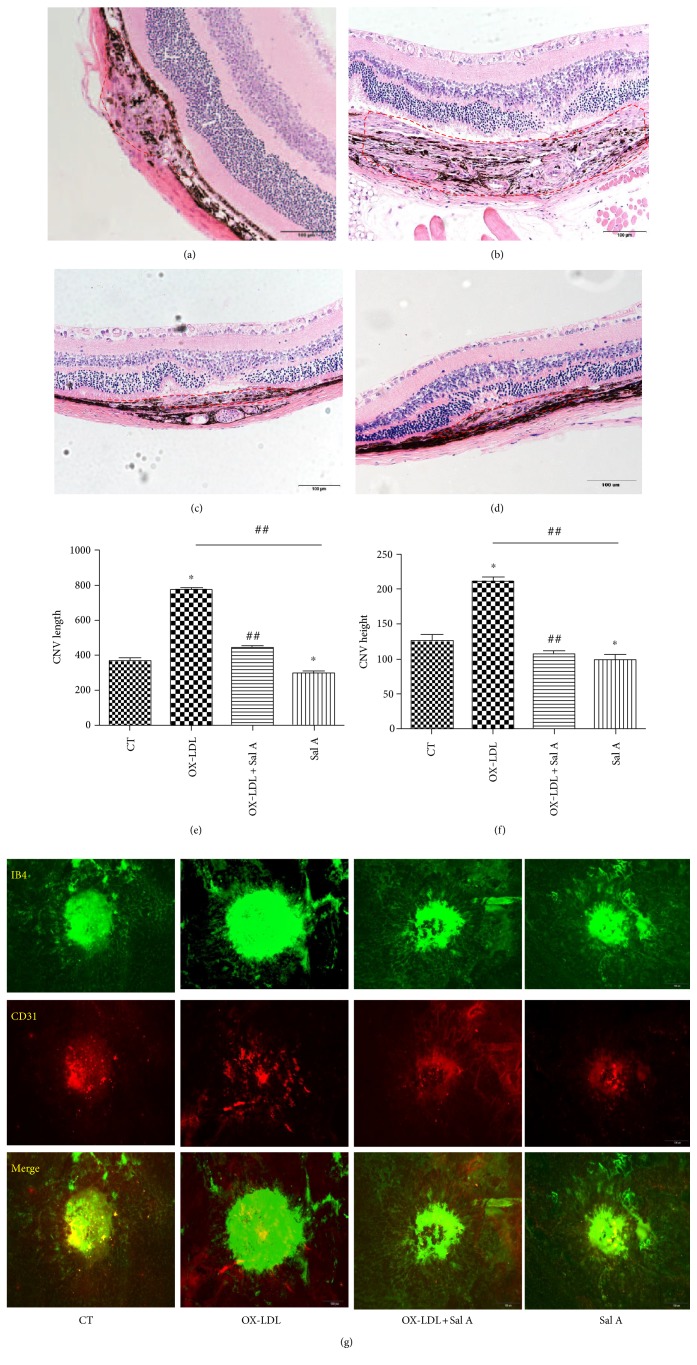
Representative images of hematoxylin and eosin staining of cross sections through choroidal neovascular lesions 7 days after photocoagulation were shown. CNV maximum thickness and length of the groups in cross sections represent as indicated: (a) control group; (b) OX-LDL group; (c) OX-LDL + Sal A group; (d) Sal A group. (e, f) CNV length and height of four groups in cross sections represent as indicated. (^∗^*P* < 0.05 versus the control group. ^##^*P* < 0.05 versus the OX-LDL group).

In morphologic cross sections, CNV in the OX-LDL group was significantly longer and higher than those in the Sal A + OX-LDL group and PBS group (length: OX-LDL 765 ± 47.1 (*μ*m) versus OX-LDL + Sal A 432.59 ± 35.1 (*μ*m) versus control 368.2 ± 27.1 (*μ*m); *P* < 0.05; height: OX-LDL 195.69 ± 16.47 (*μ*m) versus OX-LDL + Sal A 118.7 ± 16.1 (*μ*m) versus control 128.5 ± 16.9 (*μ*m); *P* < 0.05, *n* = 20 eyes/group); longer and higher CNV was also identified in the PBS group than the Sal A group (PBS length: 288.63 ± 14.1(*μ*m); height:108.97 ± 18.1(*μ*m); *P* < 0.05, *n* = 20 eyes/group) (Figure 2).

**Figure 3:**
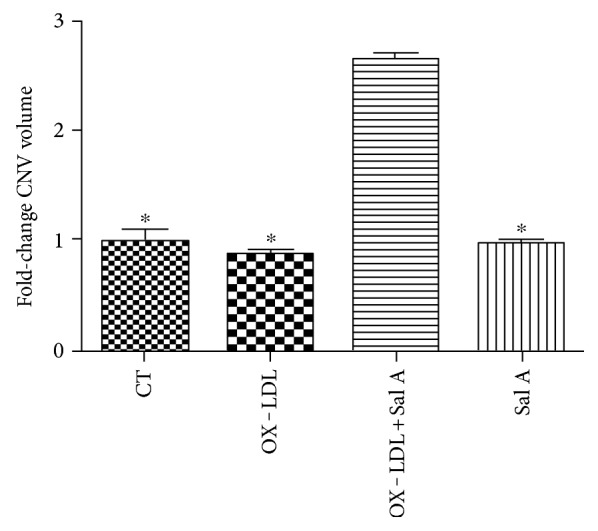
Representative images of laser CNV 7 days after photocoagulation. OX-LDL injection increased laser-induced CNV volume compared with PBS while OX-LDL + Sal A treatment reduced CNV volume compared with the OX-LDL group. Data were expressed as mean ± SEM (*n* = 20 eyes/group). ^∗^*P* < 0.05 versus the OX-LDL group. Scale bar = 100 *μ*m.

Choroidal flat mount examination showed significantly larger laser CNV volumes in the OX-LDL group compared with the PBS and OX-LDL + Sal A group, respectively (*P* < 0.01, *n* = 20 eyes/group; Figure 3()). Treatment with Sal A decreased laser CNV volumes compared with the PBS group, but the difference was insignificant (*n* = 20 eyes/group; Figure 3()).

### 4.3. Effects of OX-LDL and Sal A on Angiogenesis Gene Expression In Vivo

**Figure 4:**
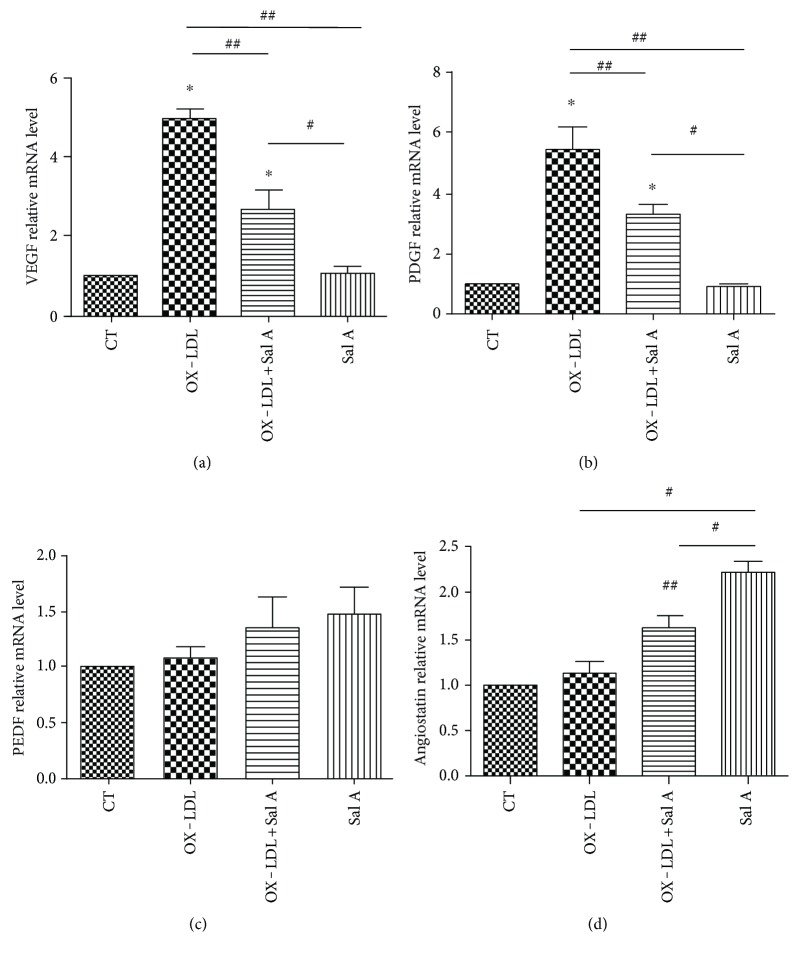
Real-time PCR analysis of VEGE, PDGF, PEDF, and angiostatin mRNA expression in four groups 7 days after laser. (a, b) OX-LDL injection increased both VEGF and PDGF mRNA expression compared with the PBS group, while OX-LDL + Sal A group reduced VEGF and PDGF mRNA expression compared with the OX-LDL group. (c) There was no difference between each group in PEDF gene expression. (d) The OX-LDL + Sal A group and Sal A group increased angiostatin gene expression compared with the PBS and OX-LDL groups. Data were expressed as mean ± SEM (*n* = 10 eyes/group). ^∗^*P* < 0.05 versus the control group. ^##^*P* < 0.05 versus the OX-LDL group. ^#^*P* < 0.05 versus the Sal A group.

The angiogenesis gene expression in RPE-choroid tissue was examined by quantitative RT-PCR 7 days after laser injury. VEGF and PDGF mRNA expression was increased in the OX-LDL-injected group when compared with the PBS group (*P* < 0.01, *n* = 10; Figures 4(a) and 4(b)), meanwhile angiostatin mRNA expression was increased in the Sal A-pretreated group (*P* < 0.05, *n* = 10; Figure 4(d)). VEGF/PDGF mRNA lvels were decreased, and angiostatin was increased in the Sal A + OX-LDL group when compared with the OX-LDL group (*P* < 0.01, *n* = 10; Figure 4(d)).There was no significant difference in PEDF mRNA level between each group (Figure 4(c)).

### 4.4. Effects of OX-LDL and Sal A on Modulating Angiogenesis Proteins In Vitro

**Figure 5:**
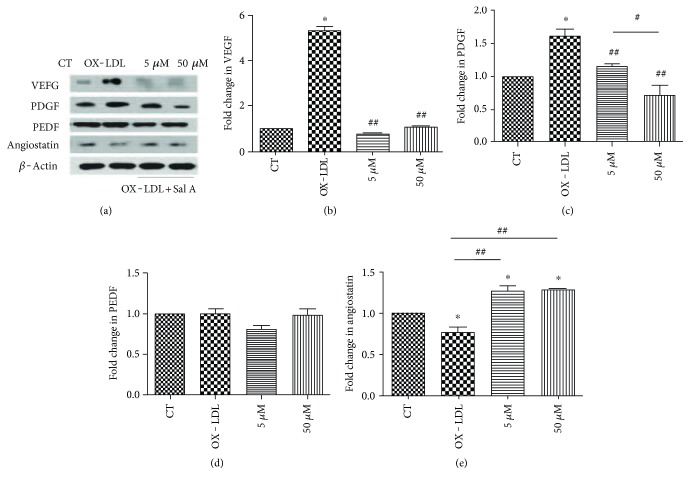
Angiogenesis proteins expression in ARPE-19 cells after OX-LDL and Sal A treatment. ARPE-19 cells were divided into control, OX-LDL(100 mg/L), and OX-LDL (100 mg/L) + Sal A (5 *μ*M/50 *μ*M) groups and cultured for 48 hours. (a) Western blot showing changes in VEGF, PDGF, PEDF, and antiangiostatin in ARPE-19 cells 48 hours after treatment. (b–e) Quantitative densitometry results showing that OX-LDL increased VEGF and PDGF levels compared with the control group, while the OX-LDL + Sal A group decreased VEGF/PDGF and slightly increased antiangiostatin level compared with the OX-LDL group. There was no significant difference between each group in PEDF level. Data were expressed as mean ± SEM. ^∗^*P* < 0.05 versus the control group. ^##^*P* < 0.05 versus the OX-LDL group. ^#^*P* < 0.05 versus the Sal A group.

In order to further explore the possible relationship between CNV progression and OX-LDL, we analyzed angiogenesis protein levels secreted by ARPE-19 cells after OX-LDL and Sal A stimulation. Western blot and ELISA were performed to evaluate VEGF, PDGF, PEDF, and angiostatin concentrations 24 and 48 hours after OX-LDL (with or without Sal A) stimulation. No significant change in any of these proteins has been detected in any of these groups at 24 hours. Comparing with the control, VEGF and PDGF were increased in the OX-LDL group, while VEGF/PDGF were decreased and antiangiostatin level was sligtly increased in the OX-LDL + Sal A (50 *μ*M) group than the OX-LDL group at 48 hours poststimulation (*P* < 0.01, Figures 5(a)–5(c) and 5(e)). There was no significant change in the PEDF level among the three groups (Figure 5(d)).

### 4.5. Effects of OX-LDL and Sal A on Tube Formation and RPE Cell Junctions

**Figure 6:**
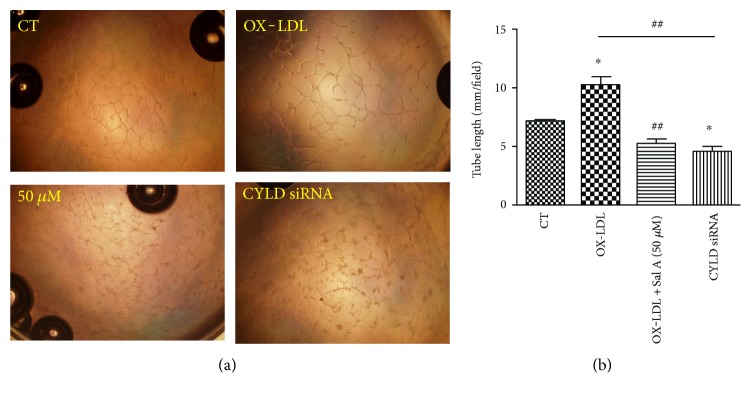
OX-LDL promotes endothelial tube formation and Sal A impairs this process. (a) HUVEC cells were plated onto matrigel and treated with SFM, OX-LDL (100 mg/L), and OX-LDL + Sal A (50 *μ*M); photographs were taken 6 hours later. Objective lens used was A-Plan 10x/0.25 NA dry (Carl Zeiss Inc.). Experiments were performed as in panel (b), and the cumulative tube length was measured. Data are expressed as the mean ± SEM; each experiments was performed in triplicate (^∗^*P* < 0.05 versus the control; ^##^*P* < 0.05 versus the OX-LDL group).

We also investigated the effects of OX-LDL and Sal A on angiogenesis process by examining vascular endothelial tube formation in vitro. Human umbilical vein endothelial cells (HUVECs) were divided into three groups: control, OX-LDL(100 mg/L), and OX-LDL + Sal A (50 *μ*M) groups. We observed capillary/tube-like structures as early as 3 hours after plating cells onto matrigel, and the structures were more evident 6 hours after plating. The tube formation was promoted in the OX-LDL group when compared with the control. This promoted tube formation induced by OX-LDL was remarkably impaired in the OX-LDL + Sal A group. By measuring the cumulative tube length, we found that OX-LDL promotes tube formation by 43% and 63%, compared with the control and OX-LDL + Sal A groups 6 hours after plating, respectively (Figures 6(a) and 6(b)).

**Figure 7:**
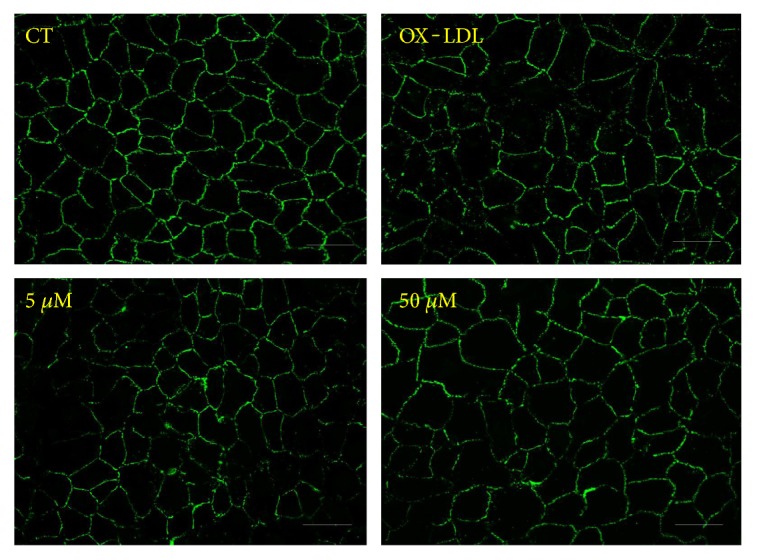
Effects of OX-LDL and Sal A on RPE cell junctions. ARPE-19 cells were classified and treated as before. ZO-1 was stained after treatment with SFM, OX-LDL (100 mg/L), and OX-LDL (100 mg/L) + Sal A (5 *μ*M/50 *μ*M) for 48 hours. Representative images of ZO-1 fluorescence showing that OX-LDL induced RPE cell junction disruption and Sal A pretreatment protected cell junction integrity. Scale bar = 50 *μ*m.

Additionally, to visualize the integrity of the RPE structure, zonula occludens-1 (ZO-1) staining was performed. Immunocytochemistry analysis revealed the more disturbed structures of RPE junctions in the OX-LDL group than the control, while Sal A pretreatment protected RPE junctions from disruption (Figure 7), suggesting that continuous treatment of OX-LDL adversely affects cell adhesion and Sal A can protect RPE cells against degeneration.

### 4.6. CYLD Involves in OX-LDL-Induced Proangiogenic Process

**Figure 8:**
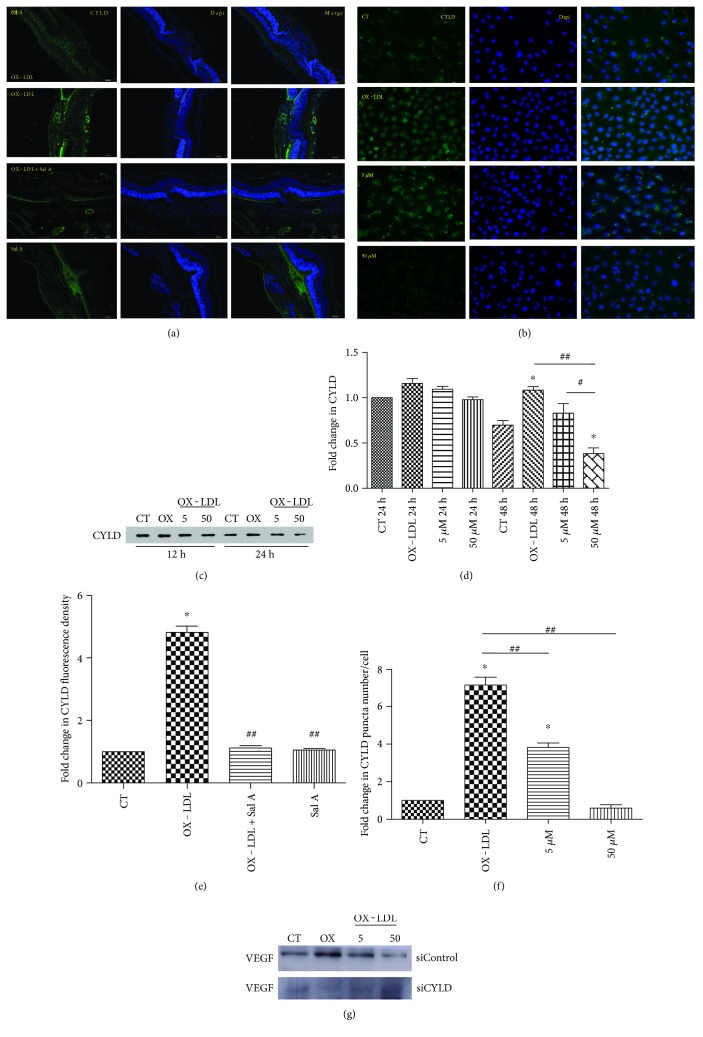
CYLD involves in OX-LDL-induced angiogenic process. C57 mice and ARPE-19 cells were treated as before. (a) Representative immunofluorescence images of CYLD in RPE/choroid of animal 7 days after laser. (b) Representative immunocytochemistry images of CYLD in ARPE-19 cells 48 hours after treatment with SFM, OX-LDL (100 mg/L), and OX-LDL (100 mg/L) + Sal A (50 *μ*M). (c, d) Western blot result of ARPE-19 cells showing that CYLD was increased in the OX-LDL group compared with the control and decreased in the OX-LDL + Sal A group compared with the OX-LDL group. (e, f) Quantitative fluorescence density results showing that CYLD fluorescence was prominent in CNV focus and higher density was found in the OX-LDL group than the control and OX-LDL + Sal A groups. (g) ARPE-19 cells were transfected with CYLD siRNA or siControl and then cultured as before for 24 hours. Western blot results showing that CYLD knockdown inhibited OX-LDL VEGF secretion. Data are expressed as the mean ± SEM (*n* = 10 eyes/group; *n* = 40 cells/group). ^∗^*P* < 0.05 versus the control group. ^#^*P* < .05 versus OX-LDL + Sal A (5 *μ*M) group after 48 hours. ^##^*P* < 0.05 versus the OX-LDL group.

Based on a previous study showing that CYLD regulates vascular endothelial cell migration and angiogenesis in HUVECs [16], we hypothesized that CYLD may also promote angiogenesis in a CNV model. Hence, we first examined CYLD in cross sections of CNV lesions by immunofluorescence. Prominent CYLD fluorescence was visualized in the retina and choroid, which was enhanced in CNV focus 7 days after laser injury (Figure 8(a)). Interestingly, fluorescence intensity in the OX-LDL + Sal A group was lower than that in the OX-LDL group, while no difference was found between the PBS and Sal A group (*P* < 0.05, *n* = 10 eyes/group, Figure 8(e)). Consistent with findings in animal experiments, immunocytochemistry and western blot results in ARPE-19 cells also showed higher CYLD expression in the OX-LDL group than control. The CYLD staining and protein expression were lower in the OX-LDL + Sal A group than OX-LDL group after culturing for 48 hours (*P* < 0.01, Figures 8(b)–8(d) and 8(f)), revealing that OX-LDL increases CYLD level and Sal A antagonizes OX-LDL by downregulating CYLD.

**Figure 9:**
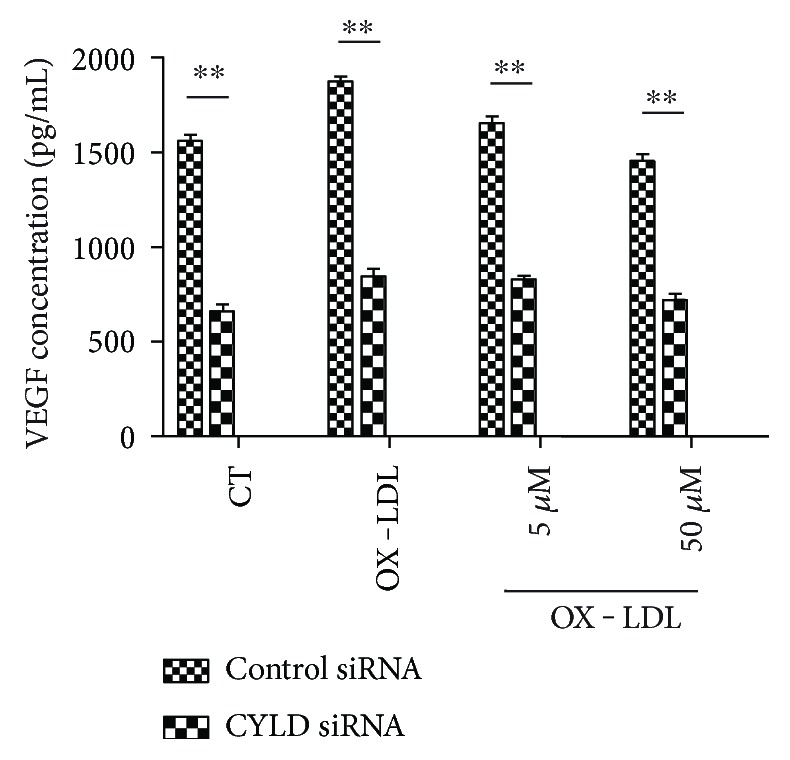
CYLD modulates VEGF level in RPE cells. ARPE-19 cells were transfected with CYLD siRNA or Control siRNA and then cultured as before for 24 hours. ELISA results showing VEGF concentrations in different groups. Data are expressed as mean ± SEM (*n* = 4). ^∗∗^*P* < 0.01 versus control siRNA.

Next, we investigated whether CYLD involves in modulating OX-LDL-induced angiogenesis process by using CYLD siRNA. As shown in Figure 9(), silencing CYLD by siRNA remarkably blocked OX-LDL-induced VEGF secretion compared with the control. Tube formation experiment also proved that silencing CYLD mRNA decreased the tube length of HUVECs after OX-LDL stimulation (Figure 6(a)).

### 4.7. Sal A Modulates CYLD via PI3K/Akt/mTOR Pathway

Our prior study demonstrated Sal A pretreatment promoted ERK and PI3K/Akt/mTOR activation in RPE cells after OX-LDL stimulation (see also data in Figure 0(a)). Thus, we tested if Sal A modulates CYLD through ERK or PI3K/Akt/mTOR pathway. As shown in Figures 0(b) and 0(c), there was no significant change of CYLD expression after the pretreatment with ERK inhibitor, but pretreatments with PI3K and mTOR inhibitor LY294002 and rapamycin markedly blocked the inhibition effect in CYLD level by Sal A, suggesting that Sal A regulates CYLD via PI3K/Akt/mTOR pathway.

### 4.8. Sal A Restrains Angiogenesis by Promoting P62-CYLD-TRAF6 Interactions

CYLD is a deubiquitinating enzyme (DUB) and recently found to physically interact with P62 and TRAF6, then negatively regulates TRAF6 function [26]. TRAF6 is a master signaling molecule controlling multiple downstream pathways. Ubiquitination of TRAF6 plays an important role in its signaling function [27]. TRAF6 ubiquitination has been demonstrated to mediate human microvascular endothelial cell sprouting [28] and cancer angiogenesis [29]. Considering that TRAF6 induces angiogenesis and TRAF6 function is regulated by CYLD, we hypothesized that Sal A may also inhibit angiogenesis by promoting CYLD-TRAF6 interaction. Coimmunoprecipitation result (Figures 1(a)–1(c)) reveals apparent P62-CYLD-TRAF6 interaction in the Sal A + OX-LDL group than that in the OX-LDL group. Sal A pretreatment inhibited ubiquitination of TRAF6, which on the on the other side is activated by OX-LDL stimulation. Collectively, the above data demonstrate that Sal A antagonizes the proangiogenesis effect of OX-LDL by decreasing CYLD level and promoting P62-CYLD-TRAF6 interaction in RPE cells.

## 5. Discussion

Previous studies have shown that oxidative stress contributes to AMD progression [30, 31]. We have demonstrated that OX-LDL induces chronic RPE cell inflammation, which is the pathogenesis of nonexudative AMD. Studies described here reveal that OX-LDL injection deteriorates laser-induced CNV progression in a mouse model. Meanwhile, OX-LDL stimulation disrupts RPE barrier and induces VEGF/PDGF secretion in ARPE-19 cells, which further indicates the pathogenic mechanism of lipid oxidation to exudative AMD. Clinical trials have revealed the association between serum lipid levels and AMD [32]. However, little is known about oxidative damage of OX-LDL to CNV pathology in vivo. We developed an animal model with high circulating serum lipoprotein level by injecting OX-LDL intravenously and verified its availability in monitoring its chronic oxidative damage to the RPE layer [10]. To our knowledge, this is the first study investigating CNV pathology using OX-LDL in animals.

Sal A is extracted from Chinese medicine *Salvia miltiorrhiza* Bunge (Danshen), which has been widely used in treating vascular diseases. Sal A scavenges free radicals and inhibits lipid and thiol peroxidation in rat liver mitochondria [33]. Hui Zhang et al. demonstrated that Sal A protects RPE cells from H_2_O_2−_-induced damage by activating Nrf2/Ho-1 [34]. In our study, Sal A has been proven to antagonize OX-LDL by reducing CNV volume and VEGF/PDGF levels. Sal A also increases antiangiostatin level, protects RPE cell junction integrity, and inhibits tube formation in HUVECs, which are inversely influenced after OX-LDL stimulation. All above evidences the inhibiting mechanism of Sal A on CNV progression.

We also demonstrated that Sal A modulates angiogenesis process by decreasing CYLD level and promoting P62-CYLD-TRAF6 interaction. CYLD is mechanistically linked to vascular endothelial barrier function [35] and anti-VEGF therapy in cancer [14]. Liu et al. [36] proved the contribution of CYLD to vascular diseases. TRAF6 also functions in promoting tumor angiogenesis [37]. Choi et al. [38] found that IL-33 induces angiogenesis and vascular leakage through TRAF6. In this research, *CYLD* gene knockdown abolished OX-LDL-induced tube formation in HUVECs and VEGF secretion in ARPE-19 cells. Sal A pretreatment inhibited OX-LDL-induced CYLD upregulation. Furthermore, the downregulation of CYLD by Sal A may be dependent on PI3K/Akt/mTOR, but not ERK. Specifically, as suggested by Jin et al. [26], deubiquitinating enzyme CYLD negatively regulated TRAF6 ubiquitination by interacting with P62 and subsequently inhibited proangiogenesis function of TRAF6. Co-IP results in our study presented that Sal A remarkably promotes P62-CYLD-TRAF6 interaction. Thus, CYLD appears to play dual roles in angiogenesis pathology: facilitating tube formation and promoting VEGF expression, meanwhile negatively regulating TRAF6 function via P62-CYLD-TRAF6 interaction.

Although our study reveals strong evidence for the pathogenic roles of OX-LDL and therapeutical effects of Sal A in age-related macular degeneration, there are still some limitations that must be addressed. First, there is a lack of direct data showing serum LDL concentration in AMD patients, especially after Sal A treatment. Moreover, further research is needed to elucidate how TRAF6 alters CNV progression and its relationship with Sal A.

In summary, our study demonstrated that increased OX-LDL level in serum has significant effects on CNV progression. Our findings also indicate pharmaceutical effects of Sal A against CNV progression. The mechanistic insights described here lay the foundation for an exciting opportunity to explore a specific concept for drug repurposing, with application to the field of exudative AMD.

## Figures and Tables

**Figure 10:**
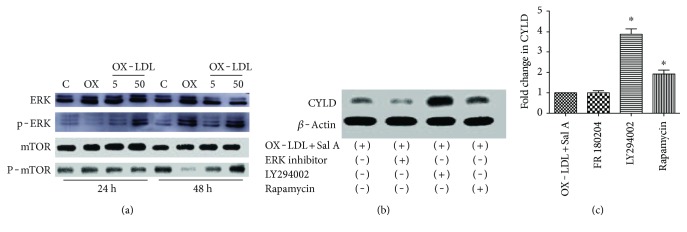
Sal A modulates CYLD via PI3K/AKT/mTOR pathway. (a) Our former study proved that Sal A promoted ERK and PI3K/AKT/mTOR phosphorylation in ARPE-19 cells after 48 hours. (b) Western blot results of CYLD showing that PI3k and mTOR inhibitor abolished Sal A-induced CYLD downregulation. (c) Quantitative densitometry results of western blot. Data are expressed as means ± ESM. ^∗^*P* < 0.05 versus the control and OX-LDL + Sal A group.

**Figure 11:**
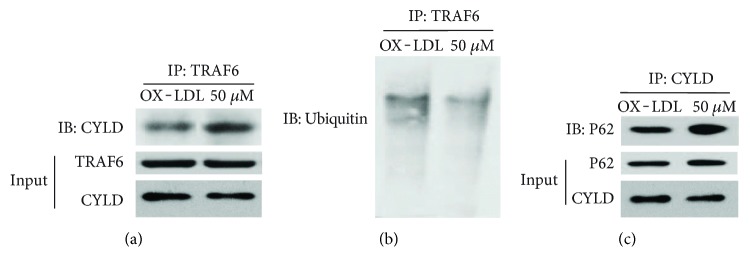
Sal A promotes P62-CYLD-TRAF6 interaction. (a) ARPE-19 cells were classified and treated as before. Coimmunoprecipitation assay was to determine P62-CYLD-TRAF6 interaction 48 hours after Sal A and/or OX-LDL stimulation. (a) TRAF6 was isolated by IP followed by detection of the associated CYLD by IB. The cell lysates were subjected to IB to monitor the expression of CYLD and TRAF6. (b)TRAF6 was isolated by IP, and its ubiquitination form was detected by IB. (c) Endogenous CYLD was isolated by IP followed by IB to detect the associated P62. P62 and CYLD protein expressions in cell lysates were monitored by direct IB. Compared to the OX-LDL group, the OX-LDL + Sal A group induced P62-CYLD TRAF6 interaction and promoted deubiquitination of TRAF6.

**Table 1:** Serum lipid levels in mouse.

Group	TC (mmol/L)	OX-LDL-C (mmol/L)
PBS	1.56 ± 0.19	0.258 ± 0.17
Sal A	1.47 ± 0.20	0.306 ± 0.11
OX-LDL	2.59 ± 0.26^∗^	0.594 ± 0.08^∗^
OX-LDL + Sal A	2.24 ± 0.17^#^	0.414 ± 0.01^#^

Values are mean ± SD; *n* = 10. ^∗^*P* < 0.05 versus the control (PBS) group; ^#^*P* < 0.05 versus OX-LDL group. TC: Total cholesterol; OX-LDL-C: Oxidized low-density lipoprotein-cholesterol.

**Table 2:** Percentages of grade 4 lesions in each group.

Groups	PBS	Sal A	OX-LDL	OX-LDL + Sal A
Rates (%)	56.25 ± 8.01	43.49 ± 3.54^∗^	66.44 ± 9.47^∗^	58.49 ± 4.47^#^

Values are mean ± SD; *n* = 10. ^∗^*P* < 0.05 significant difference when compared with the PBS group; ^#^*P* < 0.05 significant difference when compared with the OX-LDL group.

## Abbreviations

AMD: Age-related macular degeneration
CNV: Choroidal neovascularization
CYLD: Cylindromatosis
FFA: Fundus fluorescein angiography
HUVECs: Human umbilical vein endothelial cells
OX-LDL: Oxidized low-density lipoprotein
PDGF: Platelet-derived growth factor
PEDF: Pigment epithelium-derived factors
ROS: Reactive oxygen species
RPE: Retinal pigment epithelium
Sal A: Salvianolic acid A
SFM: Serum-free medium
VEGF: Vascular endothelial growth factor
ZO-1: Zonula occludens-1.
