# Corrigendum to “Salvianolic Acid A Inhibits OX-LDL Effects on Exacerbating Choroidal Neovascularization via Downregulating CYLD”

**DOI:** 10.1155/2020/1769871

**Published:** 2020-12-12

**Authors:** Ke Mao, Wanting Shu, Libin Liu, Qing Gu, Qinghua Qiu, XingWei Wu

**Affiliations:** Department of Ophthalmology, Affiliated First People's Hospital, Shanghai Jiao Tong University, Shanghai, China

In the article titled “Salvianolic Acid A Inhibits OX-LDL Effects on Exacerbating Choroidal Neovascularization via Downregulating CYLD” [1], there was inadvertent figure duplication from the authors' previous publication that presented related results [2].

It was raised to our attention that the western blots for ERK and p-ERK in Figure 10(a) in [1] are identical to those in Figure 6(a) of [2], and those for mTOR and p-mTOR in Figure 10(a) in [1] are identical to those in Figure 10(a) of [2]. Although the images represented similar experiments, the article stated “Our prior study demonstrated Sal A pretreatment promoted ERK and PI3K/Akt/mTOR activation in RPE cells after OX-LDL stimulation,” and the caption stated “Our former study proved that Sal A promoted ERK and PI3K/AKT/mTOR phosphorylation in ARPE-19 cells after 48 hours”; the other study was not cited, and the timepoints were different, i.e., 24 h and 48 h in [1] and 12 h and 24 h in [2].

The authors apologise for the error and for not citing the other article, which was not yet published when the article was submitted, and the editorial board agreed with publication of a corrigendum.

Corrected western blots for Figure 10(a) showing that Sal A promotes ERK and PI3K/AKT/mTOR phosphorylation in ARPE-19 cells after 48 hours are as follows:

## Figures and Tables

**Figure 1:**
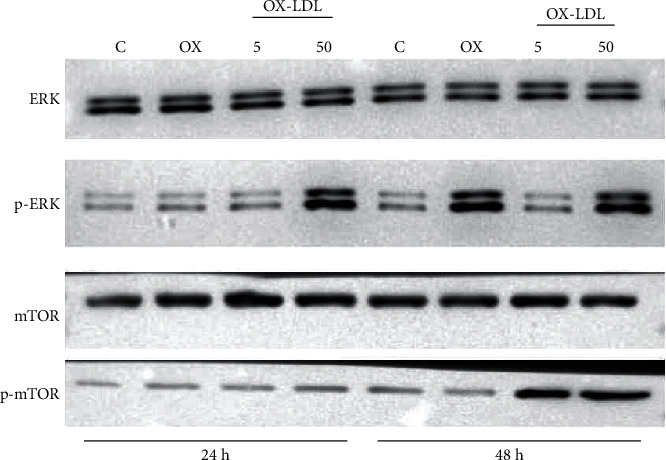
Sal A modulates CYLD via PI3K/AKT/mTOR pathway. (a) As in our former study [2], Sal A promoted ERK and PI3K/AKT/mTOR phosphorylation in ARPE-19 cells after 48 hours. From left to right: control (C), oxidized low-density lipoprotein (OX), OX-LDL plus 5 *μ*M salvianolic acid A (OX-LDL 5), and OX-LDL plus 50 *μ*M salvianolic acid A (OX-LDL 50), all for 24 hours; C, OX, OX-LDL 5, and OX-LDL 50, all for 48 hours.
